# Toyoncin, a Novel Leaderless Bacteriocin That Is Produced by Bacillus toyonensis XIN-YC13 and Specifically Targets B. cereus and Listeria monocytogenes

**DOI:** 10.1128/AEM.00185-21

**Published:** 2021-05-26

**Authors:** Juanjuan Wang, Haitao Xu, Shu Liu, Baolong Song, Hualin Liu, Feng Li, Shulin Deng, Guangli Wang, Huawei Zeng, Xin Zeng, Dayong Xu, Biao Zhang, Bingyue Xin

**Affiliations:** a College of Life Sciences, Huaibei Normal University, Huaibei, Anhui Province, China; b School of Marine Sciences, Sun Yat-sen University, Zhuhai, Guangdong Province, China; Centers for Disease Control and Prevention

**Keywords:** food preservatives, bacteriocin, *Bacillus toyonensis*, toyoncin

## Abstract

Bacteriocins have attracted increasing interest because of their potential as natural preservatives. Recent studies showed that the Bacillus cereus group is a prominent producer of bacteriocins. Using a laboratory-based screening strategy, we identified a strain in the B. cereus group, Bacillus toyonensis XIN-YC13, with antimicrobial activity against B. cereus. A novel, 70-amino-acid-long leaderless bacteriocin, toyoncin, was purified from the culture supernatant of strain XIN-YC13, and its molecular mass was found to be 7,817.1012 Da. Toyoncin shares no similarity with any other known bacteriocins, and its N-terminal amino acid is formylmethionine rather than methionine. Toyoncin shows good pH and heat stability and exhibits specific antimicrobial activity against two important foodborne pathogens, B. cereus and Listeria monocytogenes. Additionally, toyoncin exerts bactericidal activity and induces cell membrane damage. Toyoncin can also inhibit the outgrowth of B. cereus spores. Preservation assays showed that toyoncin effectively suppressed or eradicated B. cereus and L. monocytogenes in pasteurized skim milk. These results suggest that toyoncin can be used as a new biopreservative against B. cereus and L. monocytogenes in the food industry.

**IMPORTANCE** We identified a novel leaderless bacteriocin, toyoncin, produced by B. toyonensis XIN-YC13. Toyoncin shows good pH and heat stability, and it has specific antimicrobial activity against B. cereus and L. monocytogenes (two important foodborne pathogens), likely by destroying their cell membrane integrity. Toyoncin inhibited the outgrowth of B. cereus spores and effectively inhibited or eliminated B. cereus and L. monocytogenes in a milk model system. These results indicate the potential of toyoncin as a food preservative.

## INTRODUCTION

Food spoilage and foodborne pathogenic bacteria are global public concerns because they cause huge economic losses, numerous illnesses, and high mortality (1). Although chemical preservatives can inhibit or kill these pathogens and extend the storage of food products, some of these agents alter the sensory and nutritional qualities of food (benzoic acid, sorbic acid, acetic acid, lactic acid, etc.) and have adverse effects on human health (nitrite). Therefore, natural and safe food preservatives are urgently required in the food industry (2, 3).

Bacteriocins have attracted increased interest because of their potential as natural food preservatives against pathogenic and spoilage organisms (4). Bacteriocins are ribosomally synthesized peptides of bacteria that exhibit antimicrobial activities against other bacteria, either of closely related species or across genera (5). Bacteriocins have numerous desirable properties, such as their potent antimicrobial activity, pH and heat tolerance, lack of toxicity toward eukaryotic cells, sensitivity to gut proteases, and lack of interference with the sensory quality of foods, making them suitable for use in food preservation (4, 6, 7). A few bacteriocins, including nisin, pediocin, and carnocyclin A, have been commercialized as food preservatives. In particular, nisin has been approved for use as a food preservative for more than 50 years (8, 9). However, spontaneous nisin resistance has recently increased, and nisin is unstable under neutral and alkaline conditions (10–12). Studies are needed to identify novel bacteriocins that can be used along with nisin or to replace nisin to combat food spoilage and foodborne pathogenic bacteria.

The Bacillus cereus group contains spore-forming, aerobic or facultative anaerobic, rod-shaped bacteria and has multiple genetically related species (13, 14). Recent studies showed that the B. cereus group is a prominent producer of bacteriocins, and dozens of different bacteriocins, including lantibiotics, unmodified bacteriocins, sactibiotics, and circular bacteriocins, have been biochemically and genetically characterized in this group (15). Some of these show potent antimicrobial effects on a series of foodborne pathogens and food spoilage bacteria. Moreover, genome-mining studies revealed that the B. cereus group contains many different types of novel bacteriocins that have not been functionally characterized (16–18).

In this study, we purified and characterized the novel bacteriocin toyoncin, which is specifically active against B. cereus and Listeria monocytogenes, in Bacillus toyonensis strain XIN-YC13 using a culture-based bacteriocin-screening strategy. The biochemical properties and mode of action of toyoncin were evaluated, and its application potential in food preservation was investigated via growth inhibition assays of pathogenic bacteria in pasteurized skim milk.

## RESULTS

### Screening and identification of a bacteriocin-producing strain, *B. toyonensis* XIN-YC13.

**FIG 1:**
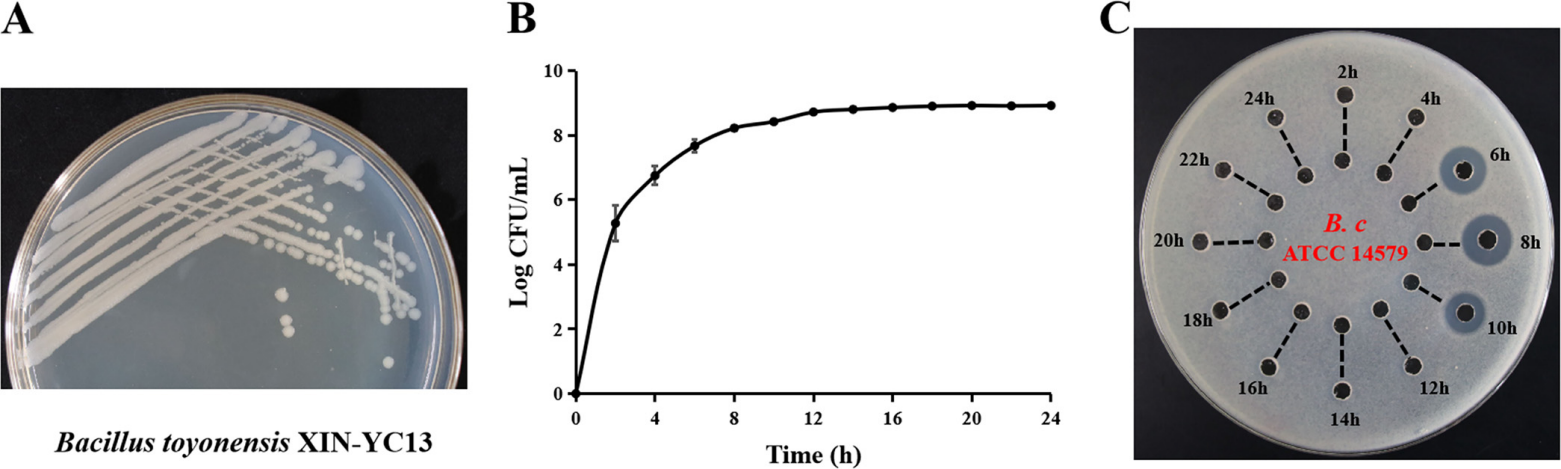
Screening the bacteriocin-producing strain Bacillus toyonensis XIN-YC13. (A) Colony morphology of B. toyonensis XIN-YC13. (B) Growth kinetics of B. toyonensis XIN-YC13 in Luria-Bertani (LB) medium. Experiments were performed in triplicate, and data are shown as mean values ± standard deviations (SD). (C) Antimicrobial activity of the supernatant of B. toyonensis XIN-YC13 against B. cereus ATCC 14579. The untreated supernatant was added to the outer well, and the corresponding supernatant treated with mixed enzymes was added to the inner well.

Using a culture-based bacteriocin-screening strategy, 102 strains from the B. cereus group were tested for their antimicrobial activity against the foodborne pathogen B. cereus strain ATCC 14579. The supernatants of 34 strains exhibited antimicrobial activity against the indicator strain (data not shown). Among them, B. toyonensis strain XIN-YC13 produced antimicrobials during the exponential growth phase (starting at 6 h; maximum antimicrobial production was recorded after 8 h, and no activity was observed after 10 h) (Fig. 1A to C). Moreover, the antimicrobial exhibited sensitivity to proteolytic digestion, suggesting that the active substance has protein/peptide properties (Fig. 1C). Strain XIN-YC13 was inferred to be a bacteriocin-producing strain and used for subsequent analysis.

### Purification and amino acid sequencing of *B. toyonensis* XIN-YC13 bacteriocin, toyoncin.

**FIG 2:**
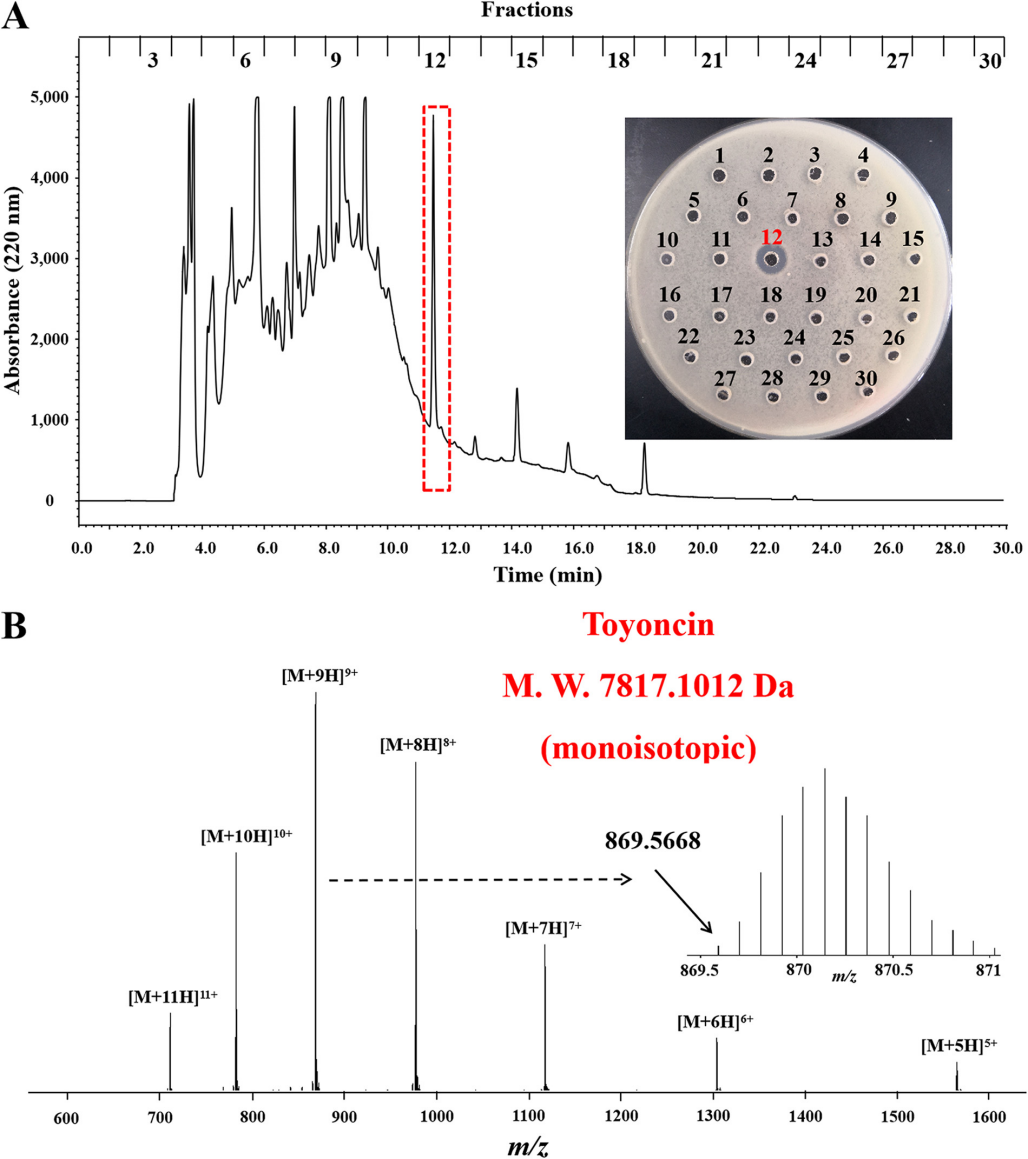
Purification and identification of toyoncin. (A) HPLC analysis of crude extracts of B. toyonensis XIN-YC13. The effluent was monitored at 220 nm and manually collected every minute. (B) Q-TOF mass spectrometry of the HPLC-purified toyoncin. M.W., molecular weight.

**FIG 3:**
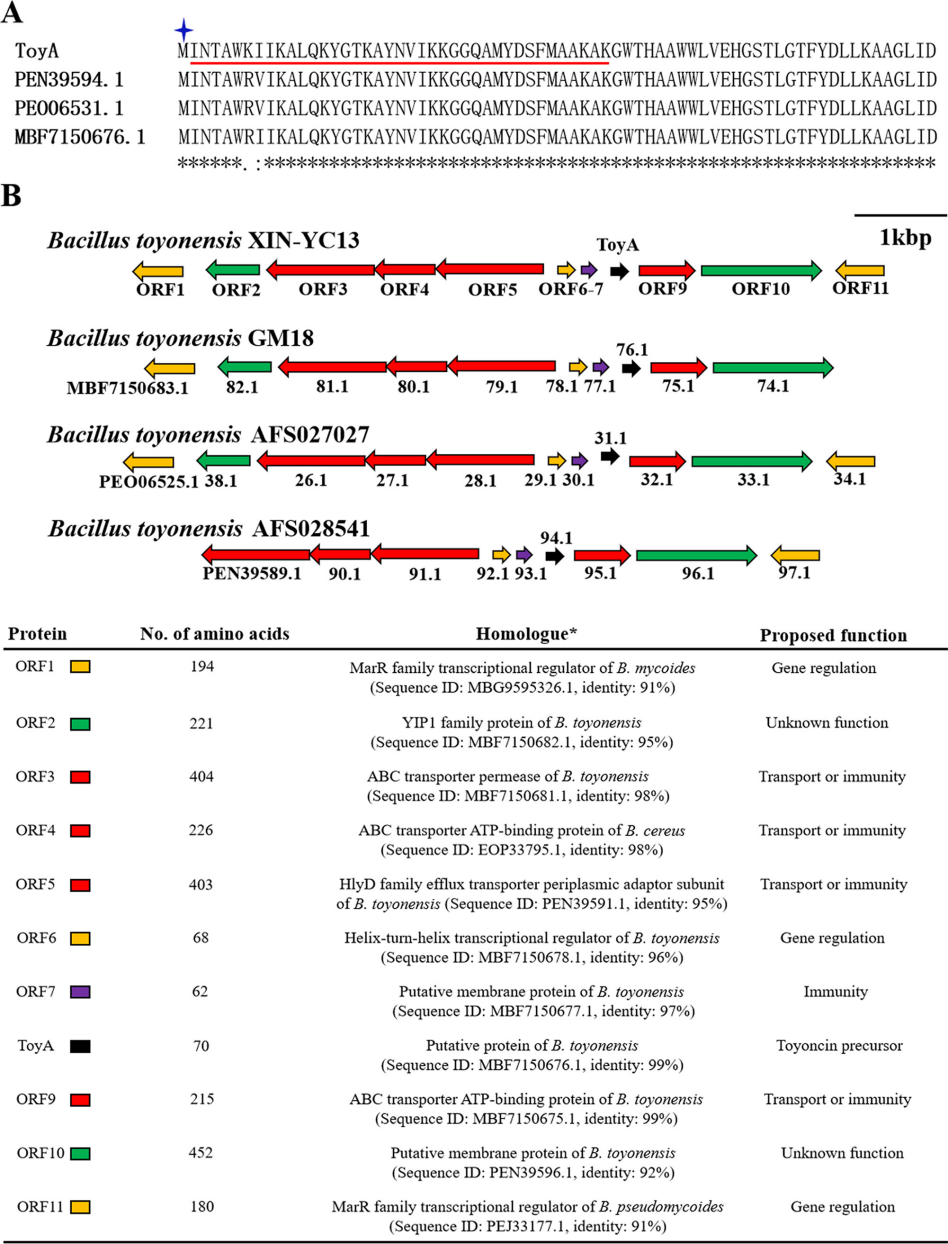
Amino acid sequences and putative biosynthetic gene clusters of toyoncin and its analogues. (A) Sequence alignment of the precursor peptide of toyoncin (ToyA) with the precursor peptides of its analogues. The underlined characters represent the 39 N-terminal amino acids of deformylated toyoncin sequenced by Edman degradation. The N-terminal amino acid of ToyA, methionine, is formylated (marked with an asterisk) to form the mature bacteriocin product toyoncin. (B) Gene clusters of toyoncin and its analogues were distributed in four Bacillus toyonensis strains. Open reading frames (ORFs) are colored according to their predicted functions. *, analyses were performed using the BLASTP program, and the proteins listed were the closest homologue to each ORF in the putative biosynthetic gene cluster of toyoncin in the GenBank database.

Bacillus toyonensis XIN-YC13 was cultured in Luria-Bertani (LB) broth for 8 h, and bacteriocin in the culture supernatants was concentrated and purified using Amberlite XAD-7 HP resin and reverse-phase high-performance liquid chromatography (RP-HPLC), respectively. As shown by the results in Fig. 2A, only fraction 12 showed antimicrobial activity against the indicator strain B. cereus ATCC 14579. The antimicrobial substance in fraction 12 was subjected to liquid chromatography-mass spectrometry (LC-MS) analysis and amino acid sequencing. The measured molecular mass of the antimicrobial substance in fraction 12, which we denoted as toyoncin, was 7,817.1012 Da (monoisotopic signal) (Fig. 2B). No sequence was obtained when toyoncin was analyzed by amino acid sequencing. This result indicates that the N terminus of toyoncin was blocked by a modification. When toyoncin was treated with cyanogen bromide, a product with a molecular mass of 7,658.0789 Da (monoisotopic signal) was generated (Fig. S1 in the supplemental material). This resulting product was approximately 159 Da less than the molecular mass of toyoncin, indicating that the first amino acid of the toyoncin precursor was a formylated methionine. The resulting product was then subjected to amino acid sequencing, and the following 39 amino acid residues were identified: INTAWKIIKALQKYGTKAYNVIKKGGQAMYDSFMAAKAK (Fig. 3A). The calculated mass of 4,507.3996 Da, which included the N-terminal formyl group (+28 Da), did not account for the molecular mass observed by MS analysis. This result suggests that the amino acid sequence of toyoncin that had been obtained was not complete.

### Primary structure and putative biosynthetic gene cluster of toyoncin.

To reveal the primary structure and genetic loci of toyoncin, the genome of B. toyonensis strain XIN-YC13 was sequenced and annotated, and all genome-encoded putative proteins were searched by the 39 amino acid residues identified as described above. A hypothetical protein that we named ToyA, comprising 70 amino acid residues, MINTAWKIIKALQKYGTKAYNVIKKGGQAMYDSFMAAKAKGWTHAAWWLVEHGSTLGTFYDLLKAAGLID, covered the above-described 39 N-terminal amino acids of treated toyoncin (underlined) (Fig. 3A). Moreover, the calculated mass of ToyA was 7,789.0606 Da, which was approximately 28 Da less than the experimentally derived mass (7,817.1012 Da), indicating that the precursor ToyA underwent formylation of the N-terminal methionine in the course of toyoncin biosynthesis. Therefore, toyoncin is a leaderless peptide comprised of 70 amino acid residues, and its N-terminal amino acid is formylmethionine rather than methionine (Fig. 3A). BLASTP analysis showed that toyoncin was a novel bacteriocin with no homology to any known bacteriocins.

The putative biosynthetic gene cluster of toyoncin, which is in the vicinity of the toyoncin structural gene, is illustrated in Fig. 3B. Upstream from *toyA*, there are seven genes (open reading frames *orf1* to *orf7*) encoding an MarR family transcriptional regulator (ORF1), a YIP1 family membrane protein (ORF2), an ABC transporter permease (ORF3), an ABC transporter ATP-binding protein (ORF4), the periplasmic adaptor subunit of the HlyD family efflux transporter (ORF5), a helix-turn-helix transcriptional regulator (ORF6), and a putative immunity protein (ORF7). Downstream from *toyA*, there are three genes (*orf9* to *orf11*) encoding an ABC transporter ATP-binding protein (ORF9), a putative membrane protein (ORF10), and an MarR family transcriptional regulator (ORF11). The gene related to the coding of formylase was not found in the vicinity of the toyoncin structural gene. In addition, B. toyonensis GM18, AFS028541, and AFS027027 all contained a highly homologous toyoncin biosynthetic gene cluster, and their precursors showed 97% (68/70) to 99% (69/70) identity to toyoncin (Fig. 3B).

### Antimicrobial activity and stability of toyoncin.

The spectrum of antimicrobial activity of HPLC-purified toyoncin was evaluated by the microdilution method. As shown by the results in Table 1, toyoncin exhibited a narrow antimicrobial spectrum and was specific against two Gram-positive species, B. cereus and L. monocytogenes (two important foodborne pathogens). The MICs of toyoncin for these pathogens were 0.78 to 3.13 μM. No other species of Gram-positive bacteria or Gram-negative bacteria tested were sensitive to toyoncin.

**TABLE 1:** Antimicrobial activities of toyoncin

Strain^*a*^	Medium^*b*^	MIC (μM)^*c*^
Gram-positive bacteria
Bifidobacterium infantis ATCC 15697	TPY	NA
Enterococcus faecalis ATCC 29212	NB	NA
Enterococcus faecalis ATCC 51299	NB	NA
Lactobacillus acidophilus GIM 1.208	MRS	NA
* *Lacticaseibacillus casei LWCC3002	MRS	NA
* *Lacticaseibacillus rhamnosus ATCC 7469	MRS	NA
* *Lactiplantibacillus plantarum LWCC3005	MRS	NA
* *Lactiplantibacillus plantarum ATCC 8014	MRS	NA
Bacillus cereus ATCC 14579	LB	0.78
Bacillus cereus CMCC 63301	LB	1.56
Bacillus cereus CMCC 63303	LB	0.78
Listeria monocytogenes LM201	TSB-YE	1.56
Listeria monocytogenes 605	TSB-YE	3.13
Listeria monocytogenes ATCC 19111	TSB-YE	1.56
Listeria monocytogenes ATCC 19115	TSB-YE	1.56
Staphylococcus aureus ATCC 43300	NB	NA
Staphylococcus aureus ATCC 29213	NB	NA
* *Staphylococcus epidermidis CMCC26069	NB	NA
Streptococcus pneumoniae ATCC 49619	BHI	NA
Streptococcus pyogenes ATCC 19615	BHI	NA
Streptococcus thermophilus CICC 6038	MRS	NA
Gram-negative bacteria
Pseudomonas aeruginosa ATCC 27853	NB	NA
Klebsiella pneumoniae CMCC46117	NB	NA
Salmonella enterica serotype Paratyphi CMCC50093	NB	NA
* Shigella* serogroups CMCC51105	NB	NA
Escherichia coli ATCC 25922	NB	NA
Acinetobacter baumannii ATCC 19606	NB	NA
* Salmonella enterica* serotype Typhimurium ATCC 14028	NB	NA
Serratia marcescens CMCC41002	NB	NA

a ATCC, American Type Culture Collection; GIM, Guangdong Institute of Microbiology; CMCC, China Medical Culture Collection; CICC, China Center of Industrial Culture Collection. *L. casei* LWCC3002 and *L. plantarum* LWCC3005 were purchased from Shanghai Luwei Microbial Sci.&Tech. Co. Ltd. (Shanghai, China). L. monocytogenes LM201 and L. monocytogenes 605 were kindly provided by Mei Liu (Huazhong Agricultural University, Wuhan, China).

b BHI, brain heart infusion broth; LB, Luria-Bertani; MRS, Li mupirocin- and cysteine hydrochloride-modified MRS medium base; NB, nutrient broth; TPY, Trypticase-Phytone-yeast extract medium; TSB-YE, tryptic soy broth with yeast extract.

c The MIC experiments were performed in triplicate; NA, toyoncin showed no activity against the indicator bacteria even at a high concentration (100 μM).

**FIG 4:**
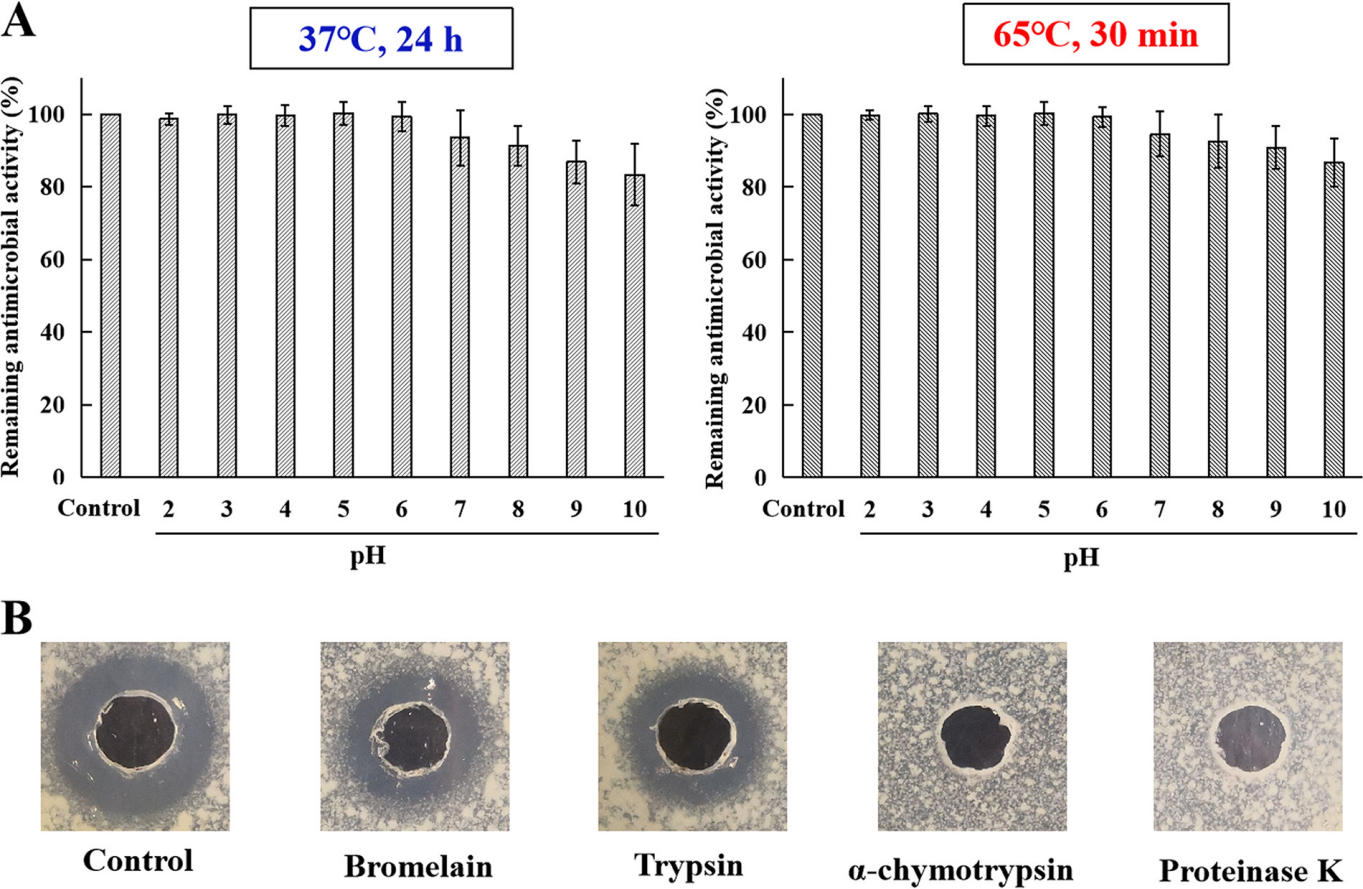
Effects of pH, temperature, and proteases on antimicrobial activity of toyoncin. (A) Stability of toyoncin at 37°C or 65°C under different pH conditions. All tests were performed in triplicate, and data are shown as mean values ± SD. (B) Effects of various proteases on toyoncin.

The susceptibility of toyoncin to pH, temperature, and proteases was investigated using well diffusion assays. Toyoncin retained 100% activity after incubation (37°C and 65°C) under acidic conditions (pH 2.0 to 6.0) (Fig. 4A). The stability of toyoncin decreased slightly under neutral and alkaline conditions (pH 7.0 to 10.0), and it retained most of its antimicrobial activity. Following incubation at pH 10.0, toyoncin maintained 83.3% (37°C for 24 h) and 86.7% (65°C for 30 min) of its activity, respectively (Fig. 4A). Toyoncin retained partial antimicrobial activity following treatment with bromelain and trypsin and completely lost its activity following digestion with α-chymotrypsin and proteinase K (Fig. 4B).

### Toyoncin exerts bactericidal activity and causes membrane damage.

**FIG 5:**
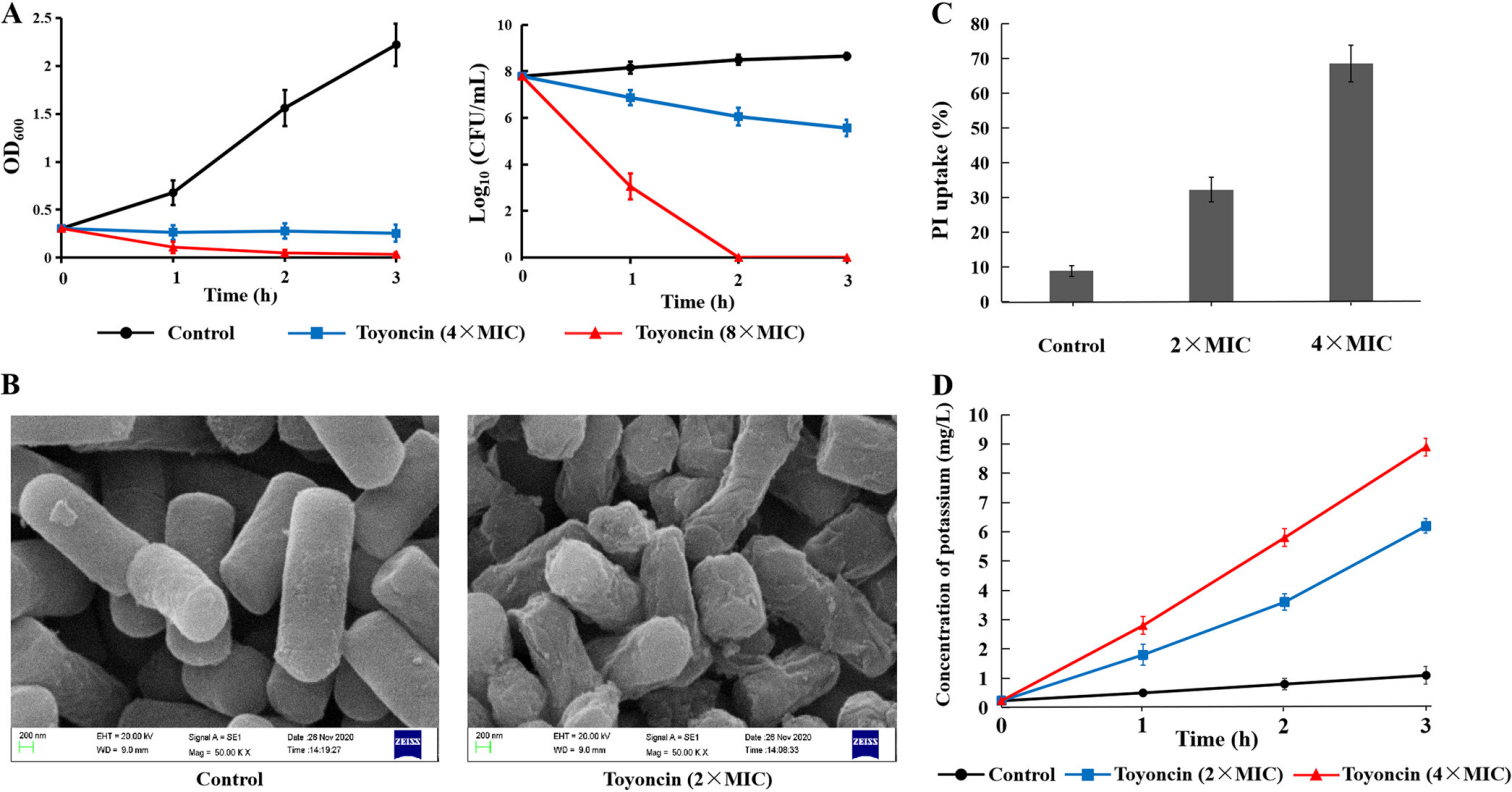
Toyoncin exerts bactericidal activity and causes membrane damage. (A) Effects of toyoncin on the number of viable cells and optical density of B. cereus ATCC 14579 culture. The data are shown as the mean values and SD. (B) SEM analysis of toyoncin-treated B. cereus cells. (C) Flow cytometry analysis of cell membrane damage by PI uptake staining. B. cereus cells were exposed to 0-, 2-, and 4-fold MIC of toyoncin for 2 h. The percentage of PI uptake by cells was analyzed with a flow cytometer. The data are shown as the mean values and SD. (D) Effect of toyoncin on amount of potassium released from B. cereus cells. The data are shown as the mean values and SD.

The bactericidal activity of toyoncin was assessed by incubation of different concentrations (4- and 8-fold MIC) of HPLC-purified toyoncin with exponential-phase cultures of B. cereus strain ATCC 14579. At a low concentration of toyoncin (4-fold MIC), the value for optical density at 600 nm (OD_600_) of the culture did not increase and the number of viable cells decreased steadily throughout the testing period (Fig. 5A); however, the OD_600_ value of the culture decreased continuously and no viable cells were detected after 2 h at a high concentration of toyoncin (8-fold MIC). These results indicate that toyoncin exerts bactericidal activity against sensitive strains.

Morphological changes in B. cereus ATCC 14579 cells after treatment with toyoncin were observed by scanning electron microscopy (SEM). As shown in Fig. 5B, untreated B. cereus cells showed a regular, smooth, and intact cell surface; in contrast, toyoncin treatment (2-fold MIC) caused significant membrane damage and the cell surface appeared rough, collapsed, and deformed. The cell membrane integrity of B. cereus cells postincubation with toyoncin was evaluated in a propidium iodide (PI) uptake assay and a potassium efflux assay. As shown by the results in Fig. 5C, the proportions of PI-stained B. cereus cells increased markedly, to 32.3% (2-fold MIC) and 68.6% (4-fold MIC), compared to the percentage in the control (8.9%). Toyoncin also caused time- and dose-dependent increases in potassium release from B. cereus cells (Fig. 5D). After treatment with toyoncin for 3 h, the concentrations of potassium in the supernatants increased significantly, to 6.2 mg/ml (2-fold MIC) and 8.9 mg/ml (4-fold MIC), compared to the concentration in the control (1.1 mg/ml). These results strongly support that toyoncin is a bacteriocin that kills sensitive bacteria by causing cell membrane damage.

### Toyoncin inhibits the outgrowth of B. cereus spores.

**FIG 6:**
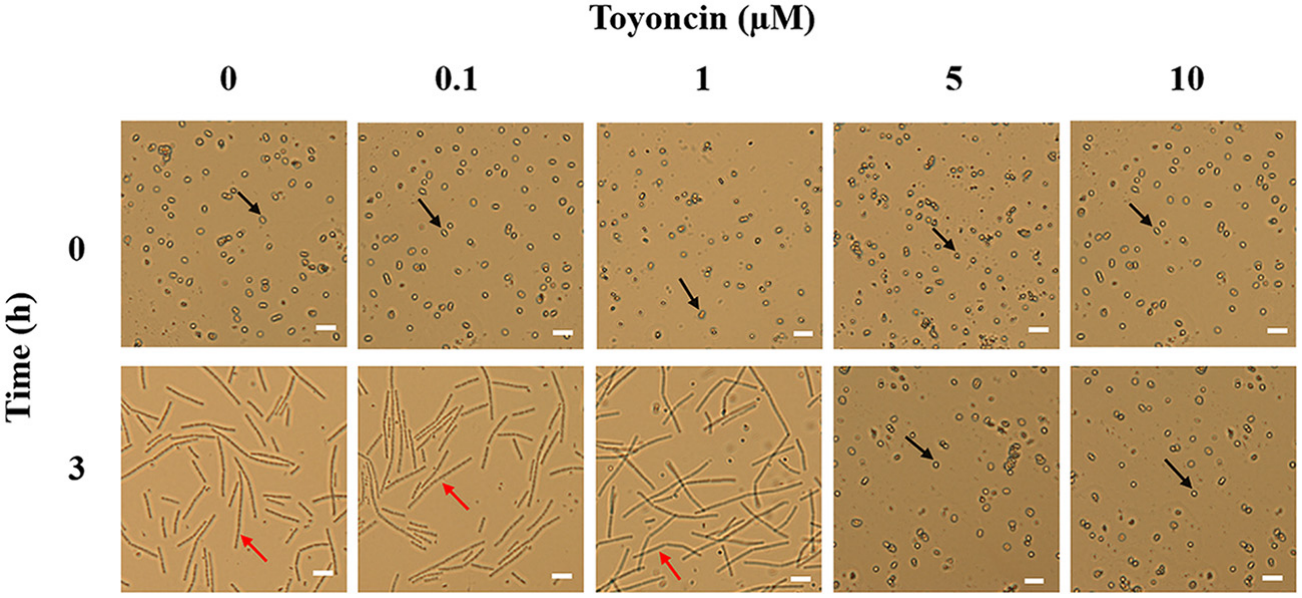
Toyoncin inhibits the outgrowth of Bacillus cereus spores. Black arrows indicate spores of B. cereus ATCC 14579. Red arrows indicate vegetative cells of B. cereus ATCC 14579. Scale bars represent 5.0 μm.

To determine the capability of toyoncin to prevent spore outgrowth, B. cereus ATCC 14579 was selected as a model. As shown in Fig. 6, when the culture contained low concentrations of toyoncin (0.1 or 1 μM), the spores germinated and transformed into chains of vegetative bacilli after incubation for 3 h. When high concentrations of toyoncin were added to the culture (5 or 10 μM), spore outgrowth was inhibited and no bacilli were observed. These results demonstrate that toyoncin can inhibit B. cereus spore outgrowth at concentrations above 5 μM.

### Preservative effect of toyoncin against foodborne pathogens in milk samples.

**FIG 7:**
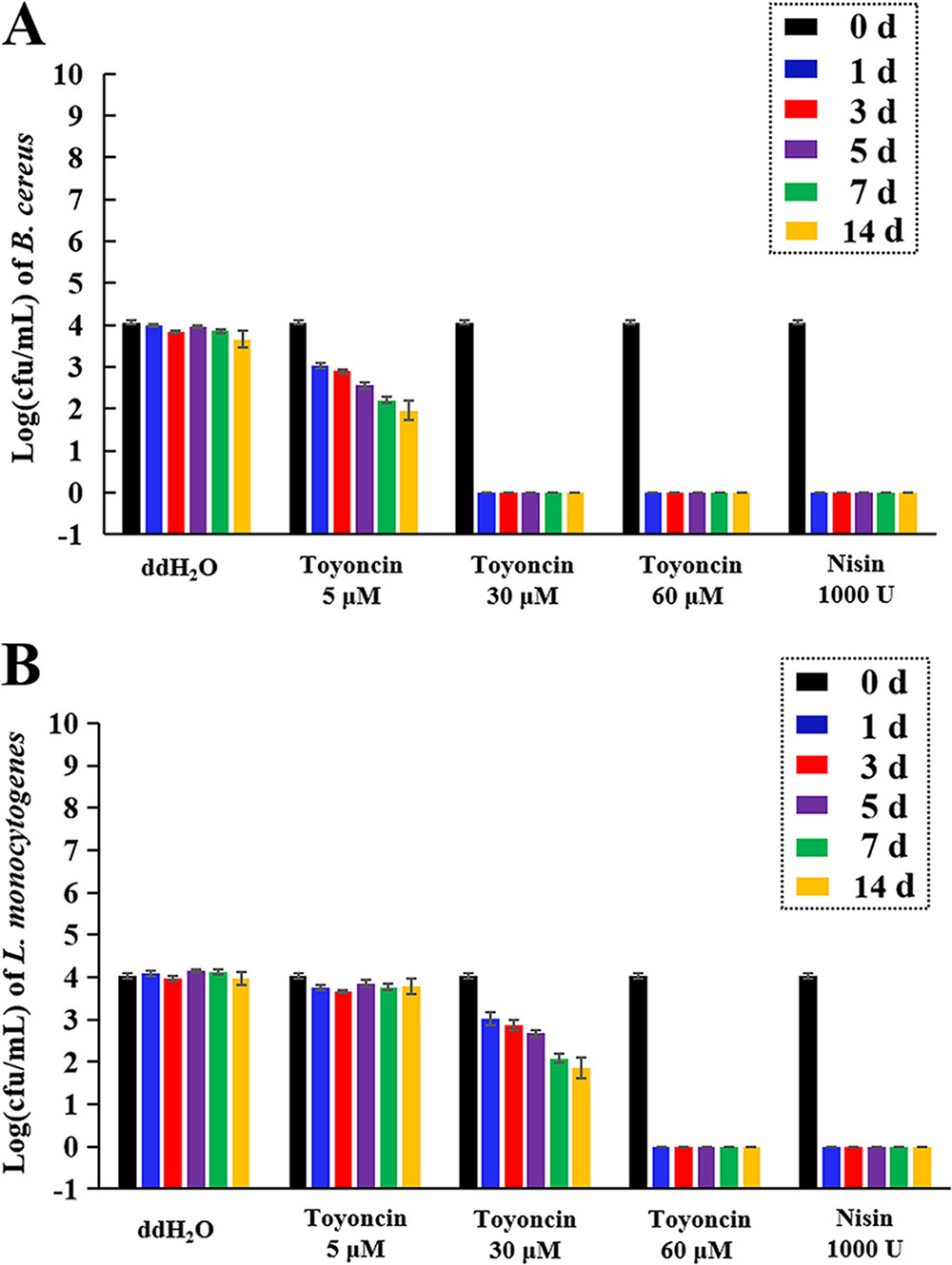
Preservative effect of toyoncin against B. cereus (A) and L. monocytogenes (B) in pasteurized skim milk. Pasteurized milk samples with B. cereus and L. monocytogenes were stored at 4°C for 14 days. Commercial nisin A (1,000 U) was used as a positive control, and ddH_2_O was used as a negative control. All tests were performed in triplicate, and data are presented as mean values ± SD.

The preservation effect of toyoncin against B. cereus and L. monocytogenes was evaluated in pasteurized skim milk. When the milk samples were supplemented with low concentrations of toyoncin (5 μM) and incubated at 4°C, only B. cereus growth was significantly inhibited during the storage period, with a difference of 1.69 ± 0.08 log CFU/ml between cells treated with the negative control (double-distilled water [ddH_2_O]) and with toyoncin on day 14 (Fig. 7A). When 30 μM toyoncin was added to the milk samples and incubated at 4°C, all B. cereus cells were killed on the first day (Fig. 7A); the growth of L. monocytogenes was significantly inhibited during the storage period, with a difference of 2.13 ± 0.07 compared to the growth of cells treated with ddH_2_O on day 14 (Fig. 7B). When the concentration of toyoncin was increased to 60 μM, its preservation effect was the same as that of the positive control, nisin (1,000 U), and all B. cereus and L. monocytogenes cells were killed on the first day (Fig. 7A and B). These results suggest that toyoncin can effectively inhibit or eradicate B. cereus and L. monocytogenes in pasteurized skim milk in a concentration-dependent manner and that the preservation effect against B. cereus is better than that against L. monocytogenes.

## DISCUSSION

The increasing demand for food products with a long shelf-life and for minimally processed foods or “fresh foods” free from chemical preservatives has compelled food industries to utilize natural preservatives, such as bacteriocins (4, 6). In the last few decades, the research and application of bacteriocins have focused on bacteriocins from lactic acid bacteria; the most representative bacteriocin is the lactibiotic nisin (19). Nisin is the first and most widely used bacteriocin in food and has been approved for use as a food preservative for over 50 years (8, 9). However, the emergence of strains resistant to nisin and limited stability of nisin under neutral and alkaline conditions have stimulated the search for new bacteriocins with improved physicochemical properties and potencies (10–12). This study reports a novel leaderless bacteriocin, toyoncin, that was produced by B. toyonensis strain XIN-YC13 and whose specific antimicrobial activity against B. cereus and L. monocytogenes (two important foodborne pathogens) and high pH and thermal stability highlight its potential as a new biopreservative.

Alvarez-Sieiro et al. (19) proposed dividing bacteriocins into three classes according to their biological activities and biosynthesis mechanisms. Class I bacteriocins are ribosomally produced and posttranslationally modified peptides and are smaller than 10 kDa. Class II bacteriocins are unmodified peptides with molecular masses of <10 kDa and comprise four subclasses: pediocin-like (class IIa), two-peptide (class IIb), leaderless (class IIc), and non-pediocin-like single-peptide (class IId) bacteriocins. Class III bacteriocins are thermolabile and larger than 10 kDa. According to these classification criteria, toyoncin belongs to the class IIc bacteriocins, or leaderless bacteriocins. More than a dozen leaderless bacteriocins have been reported, and they have been divided into three groups (single-peptide, two-peptide, and multipeptide bacteriocins) (20). Based on their amino acid sequence homology, the single leaderless bacteriocins were divided into three subgroups (aureocin A-53-like, LsbB-like, and EntL50-like). Toyoncin is a single leaderless bacteriocin but shows no similarity to any other known bacteriocins. Therefore, it can be recognized as a novel subgroup of single leaderless bacteriocins. In addition, leaderless bacteriocins are often relatively small (26 to 53 amino acids), and toyoncin is the longest among identified leaderless bacteriocins, with 70 amino acids (20, 21).

Most bacteriocins are derived from inactive precursor peptides, which include an N-terminal leader peptide and a C-terminal core peptide (5, 22). Leaderless bacteriocins have no N-terminal leader peptides and are active immediately after translation (20). Compared with other types of bacteriocins, this simplicity in the biosynthesis of leaderless bacteriocins is their most remarkable advantage, and their scale-up production can be adapted to any expression system. Considering the presence of formylated methionine at the N terminus of toyoncin, we will perform chemical synthesis to produce the toyoncin peptide with nonformylated methionine and test the effect of formylated methionine on its antimicrobial activity. The N-terminal formylation is also present in other leaderless bacteriocins, such as lacticin Z, lacticin Q, and aureocin A53, and their corresponding nonformylated N-terminal methionine peptides have comparable antimicrobial activities (20, 23). If formylated methionine has no negative effect on the antimicrobial activity of toyoncin, we will attempt to express toyoncin heterologously in Escherichia coli, Bacillus subtilis, or a yeast recombinant expression system to enhance the yield of toyoncin for further applications.

The kinetics of the bacteriocin production assay indicated that XIN-YC13 produced toyoncin during the exponential growth phase, and antimicrobial activity was detected only at 6, 8, and 10 h (Fig. 1). The production of many bacteriocins in the B. cereus group was similar to that of toyoncin, such as thuricin 4A-4, ticin, thusin, cerecyclin, and bacicyclicin XIN-1, and their antimicrobial activities were detected only in the exponential growth phase but not after this phase (15, 17, 18, 24, 25). This may be attributed to two factors: first, these bacteriocins’ gene clusters were only transcribed during the exponential growth phase, which was confirmed in a previous study of thuricin 4A-4 (18), and second, bacteriocin-producing strains secreted some proteases during their growth, which could degrade the synthesized bacteriocins. When bacteriocins were no longer synthesized and synthesized bacteriocin was decreased or had even disappeared because of enzymolysis, their antibacterial activity was not detected after the exponential growth phase. This hypothesis should be verified in further studies.

Whole-genome sequencing and DNA sequence analysis of B. toyonensis XIN-YC13 revealed the putative biosynthetic gene cluster of toyoncin (Fig. 3B). This gene cluster contained genes coding for the toyoncin precursor, membrane proteins with unknown function, ABC-type transporter-related proteins that may be responsible for the immunity and/or secretion of toyoncin, and multiple transcription regulators. Sequence homology analysis showed that the toyoncin or toyoncin-like gene cluster was present in only a few B. toyonensis strains. This suggests that toyoncin is a unique bacteriocin of B. toyonensis and that its gene cluster underwent horizontal transfer in the species during evolution. In addition, similar to the reported biosynthetic gene clusters of N-terminally formylated leaderless bacteriocins (lacticin Q, lacticin Z, and aureocin A53), the gene related to formylase synthesis was also not found in the vicinity of the toyoncin structural gene, suggesting that the toyoncin precursor underwent N-terminal formylation by a host-encoded formylase which existed outside the toyoncin biosynthetic gene cluster (20, 26–28).

Toyoncin exhibited a narrow antimicrobial spectrum, with antimicrobial activity against B. cereus strains and L. monocytogenes strains (Table 1). Remarkably, toyoncin also was inactive toward some common probiotics (Bifidobacterium infantis, Lactobacillus acidophilus, Lacticaseibacillus casei, Lacticaseibacillus rhamnosus, Lactiplantibacillus plantarum, and Streptococcus thermophilus), even at a high concentration (100 μM). Compared to other broad-spectrum bacteriocins, including nisin, the specific antimicrobial activity of toyoncin is advantageous, particularly in fermented foods (29). Toyoncin can be used to specifically target B. cereus strains and L. monocytogenes strains and has no effect on other, desirable microorganisms present in food products. Moreover, analysis of whether toyoncin could suppress the proliferation of pathogens in pasteurized skim milk showed that toyoncin effectively inhibited or eradicated B. cereus and L. monocytogenes (Fig. 7). However, the amount of toyoncin used in the milk samples must be greater than that in the culture medium to achieve the same preservative effect. It was reported that the efficacy of a bacteriocin in foodstuff is closely related to the physical and chemical properties of the food (pH, proteases, additives, etc.) and that bacteriocins may be inactivated by, precipitated by, or interact with certain food components, thus limiting their antimicrobial activity (4). We will determine the factors affecting the efficacy of toyoncin to develop approaches for maximizing its efficacy in foods in our further studies.

The stability of toyoncin under acidic or basic and heat conditions was tested; toyoncin was stable under acidic conditions and exhibited a slight loss of activity under neutral and alkaline conditions (Fig. 4). Many bacteriocins, including nisin, are readily inactivated under neutral and alkaline conditions, whereas toyoncin showed higher stability against pH and heat. Therefore, the extraordinary stability of toyoncin is another desirable property supporting its use in the food industry, because it can be applied widely, not only in acidic food products but also in neutral or alkaline food products.

We used various methods to determine the mode of action of toyoncin. According to the growth and time-kill curve assays, toyoncin exerted bactericidal activity against sensitive cells of B. cereus ATCC 14579 (Fig. 5A). Subsequent SEM analysis clearly showed that sensitive cells treated with toyoncin exhibited obvious morphological changes (Fig. 5B). PI is a nucleic acid stain that can only cross an impaired cell membrane, and the PI uptake assay showed that PI entered cells treated with toyoncin, demonstrating that toyoncin caused cell membrane damage (Fig. 5C). The increase in the extracellular potassium concentration confirmed the damage to the cell membrane of the sensitive strain (Fig. 5D). Based on these results, toyoncin kills indicator strains by damaging the integrity of the cell envelope.

Metabolically dormant *Bacillus* and *Clostridium* spores are common contaminants of food products (30). They are metabolically inactive and dehydrated and, thus, can resist some extreme conditions (wet and dry heat, UV radiation, toxic chemicals, etc.); their control and eradication are challenges in the food industry (31–33). High heat alone or in combination with chemical additives is generally used to control these spores, but extreme treatments alter the nutritional value and flavor of food and may adversely affect human health (30). The use of a bacteriocin to control and eradicate spores is considered a safe and effective method. In this study, we found that toyoncin not only effectively kills vegetative cells of B. cereus but also inhibits the outgrowth of B. cereus spores. Therefore, toyoncin has potential as a new biopreservative to protect consumers from the harmful, pathogenic, spore-forming B. cereus.

## MATERIALS AND METHODS

### Screening for bacteriocin-producing strains.

We tested the antimicrobial activities of 102 strains from the B. cereus group against B. cereus ATCC 14579. These strains were isolated from the soil in Anhui Province in China and preserved in our laboratory. Each strain was activated overnight in LB medium, and the culture (1 ml) was transferred into LB medium (100 ml) and incubated for 24 h (30°C). Cell growth was monitored every 2 h by measuring the numbers of viable cells on LB agar plates. The antimicrobial activity of the supernatants (2 h to 24 h) was assayed by the agar well diffusion method (34). Briefly, the indicator strain, B. cereus ATCC 14579 (1.0 × 10^6^ CFU/ml) was spread onto 20-ml LB agar plates (∼45°C). Wells were punched, and 50 μl of cell-free supernatant of each isolate was added to the wells. The plates were incubated at 4°C for 2 h and then at 30°C for 16 h, after which the inhibition zone was measured to assess antimicrobial activity. Cell-free supernatants with antimicrobial activities were incubated with mixed proteases at 37°C for 3 h. The mixed proteases contained trypsin and α-chymotrypsin at concentrations of 1 mg/ml. Residual antimicrobial activity was measured using the agar well diffusion method as described above. The cell-free supernatants of B. toyonensis strain XIN-YC13 showed potent antimicrobial activity against B. cereus ATCC 14579, and the antimicrobials were degraded by proteolytic enzymes. These results suggested that B. toyonensis XIN-YC13 is a bacteriocin-producing strain, and thus, it was selected for further analysis.

### Purification of bacteriocin of strain XIN-YC13.

The XIN-YC13 strain was cultured in LB broth (3 L) for 8 h (OD_600_ = 3.0), after which the cell-free supernatant was obtained by centrifugation (10,000 × *g* for 5 min at 4°C). Antimicrobial substances in the supernatants were concentrated using Amberlite XAD-7HP resin (Sigma, St. Louis, MO, USA). Briefly, 3 L of the cell-free supernatant was shaken with 300 g of resin for 12 h (4°C). The resin was poured into a column and sequentially washed with 2 L of distilled water and 1 L of 30% (vol/vol) ethanol. The antimicrobials were eluted with 500 ml of 80% (vol/vol) ethanol (pH 2.0). The eluate was condensed using a rotary evaporator, and the concentrate generated (5 ml, approximately) was centrifuged (10,000 × *g* for 5 min). The resulting supernatant was designated the crude extract (CE) and used in subsequent purification.

Reverse-phase high-performance liquid chromatography (RP-HPLC; Dionex Ultimate 3000 system, Sunnyvale, CA, USA) was used to further purify the CE of XIN-YC13. The eluted solvent contained ddH_2_O with 0.1% trifluoroacetic acid and acetonitrile. The CE (50 μl) was loaded into an Agilent HC-C_18_(2) column and separated over a linear gradient of 10 to 90% acetonitrile within 30 min. The flow rate of the mobile phase was 1.0 ml/min, and the effluent was manually collected every minute. Using the agar well diffusion method, the antimicrobial activity of each fraction was tested. The fraction showing antimicrobial activity was manually collected 100 times. The collected sample was lyophilized, and the powder generated was dissolved in ddH_2_O to prepare a stock solution at a concentration of 1 mM. The purified bacteriocin of XIN-YC13 was designated toyoncin.

### N-terminal amino acid sequencing and LC-MS analysis of toyoncin.

The Agilent 6540 ultra-high-definition (UHD) accurate-mass quadrupole time of flight (Q-TOF) LC-MS system was used for MS analysis of toyoncin. The conditions for MS operation were as follows: the ionization mode was positive, source voltage was 3.5 kV, nebulizer pressure was 35 lb/in^2^ on the gauge, drying gas flow was 9 L/min, and capillary temperature was 350°C. N-terminal amino acid sequencing of toyoncin was performed using a PPSQ-33A protein sequencer (Shimadzu, Kyoto, Japan) at Beijing Tailian Biotechnology Co., Ltd. (Beijing, China). Deformylation of toyoncin was performed as described previously (26). Briefly, toyoncin and cyanogen bromide were dissolved in 70% (vol/vol) formic acid with a molar ratio of 1:100, and the reaction was performed at 30°C for 24 h. The product generated was used for amino acid sequencing and mass spectrometry analysis as described above.

### Genome sequencing and sequence analysis of *B. toyonensis* XIN-YC13.

The genome of strain XIN-YC13 was sequenced on an Illumina HiSeq 2500 platform (San Diego, CA, USA) to a final coverage of 200-fold. The paired-end-read quality control, genome assembly, and annotation were performed with FASTP, SPAdes, and Prokka integrated in PGCGAP, respectively (35). Functional analysis of proteins was performed using the BLASTP program. Membrane proteins were predicted using the TMHMM Server, version 2.0 (36). Protein sequence alignment was performed using ClustalW (37).

### MICs of toyoncin.

The MICs of toyoncin for the indicator strains (Table 1) were determined by the microdilution method (38). Briefly, 50 μl of the indicator strain culture (1 × 10^6^ CFU/ml) and 50 μl of 2-fold serial dilutions of toyoncin were added to 96-well plates. The plates were incubated at an appropriate temperature for 20 h, and the absorbances of the mixed solutions were assayed using a microplate reader at 600 nm. The MIC was defined as the lowest concentration that visibly inhibited growth. The MIC experiments were performed in triplicate.

### Growth and time-kill curves.

HPLC-purified toyoncin was incubated with B. cereus ATCC 14579 cultures (OD_600_ = 0.3) at final concentrations of 0-, 4-, and 8-fold MIC for 3 h. Cell growth was monitored hourly by measuring the numbers of viable cells on LB agar plates and OD_600_ values of the cultures. All experiments were performed in triplicate.

### Propidium iodide uptake.

HPLC-purified toyoncin was incubated with B. cereus ATCC 14579 cultures (OD_600_ = 0.5) at final concentrations of 0-, 2-, and 4-fold MIC for 2 h at 30°C. The cells were harvested by centrifugation (6,000 × *g* for 10 min), washed three times with phosphate-buffered saline (PBS) (0.1 M, pH 7.2), and resuspended in the same PBS. The bacterial suspensions were incubated with 10 μg/ml PI (Sigma) for 30 min at 30°C. A BD FACSCalibur flow cytometer (BD Biosciences, Franklin Lakes, NJ, USA) was used to analyze the percentages of PI uptake by cells at 488-nm excitation. All experiments were performed in triplicate.

### Potassium efflux.

Bacillus cereus ATCC 14579 was cultured overnight at 30°C and then harvested, washed, and resuspended in normal saline (OD_600_ = 0.5). Next, 1-ml amounts were incubated with toyoncin (2- and 4-fold MIC) at 30°C for different times (1, 2, and 3 h). The amounts of K^+^ released were measured using an Agilent 5100 ICP-MS (Agilent Technologies, Santa Clara, CA, USA). All experiments were performed in triplicate.

### Scanning electron microscopy.

HPLC-purified toyoncin was incubated with B. cereus ATCC 14579 (OD_600_ = 0.3) to final concentrations of 0- and 2-fold MIC. After 3 h, the cells were obtained by centrifugation (6,000 × *g* for 10 min), washed three times with 0.1 M PBS (pH 7.2), and fixed with 2.5% (vol/vol) glutaraldehyde for 12 h at 4°C. The cells were dehydrated with gradient alcohol solutions (10%, 30%, 50%, 70%, 90%, and 100%) for 20 min, treated with isoamyl acetate for 20 min, dried in a vacuum freeze dryer for 12 h, and coated with gold-palladium for visualization with a Zeiss Sigma 300 scanning electron microscope (Carl Zeiss NTS, Oberkochen, Germany).

### Sensitivity to pH, temperature, and proteases.

The effect of pH and temperature on toyoncin was investigated by adjusting the pH of toyoncin solution (8-fold MIC) from 2.0 to 10.0 with 1 M HCl or 1 M NaOH solution, followed by incubation at 65°C for 30 min or 37°C for 24 h. Untreated toyoncin solution was used as a control. All experiments were performed in triplicate. The effects of various proteases on toyoncin were also tested. The proteases included bromelain (300 U/mg), trypsin (≥10,000 U/mg), α-chymotrypsin (≥40 U/mg), and proteinase K (≥40 U/mg), and each protease solution (2 mg/ml) and toyoncin solution (16-fold MIC) was mixed in equal volumes and incubated at 37°C for 1 h. The remaining activity of treated toyoncin solution was determined by the agar well diffusion method as described above. The indicator strain in this experiment was B. cereus ATCC 14579.

### Inhibition of spore outgrowth by toyoncin.

To determine whether toyoncin can inhibit B. cereus spore germination, an assay was performed as described previously (15). Briefly, 100 μl of 1.0 × 10^7^ spores/ml of B. cereus, 800 μl of LB medium, and 100 μl of toyoncin solutions at different final concentrations (0.1, 1, 5, and 10 μM) were mixed and incubated at 30°C for 3 h. Differential interference contrast microscopy images of vegetative cells or spores in the cultures were obtained with a DeltaVision epifluorescence microscope (Applied Precision, Issaquah, WA, USA), and the images were processed with the SoftWoRX Explorer Suite program (Applied Precision).

### Applications of toyoncin in the preservation of pasteurized skim milk.

Pasteurized skim milk was inoculated with B. cereus and L. monocytogenes, and different concentrations of toyoncin were used to evaluate its antibacterial potential. Briefly, B. cereus ATCC 14579, B. cereus strain CMCC63303, L. monocytogenes strain LM201, and L. monocytogenes strain 605 were all cultured overnight and diluted to 1 × 10^5^ CFU/ml. The diluted cultures of the four strains were mixed with equal volumes of the prepared mixed culture. Next, 100 μl of mixed cultures of B. cereus and L. monocytogenes, 800 μl of pasteurized skim milk, and 100 μl of toyoncin solutions at different final concentrations (5, 30, and 60 μM) were mixed and incubated at 4°C for 14 days. The numbers of viable bacterial cells in the milk samples were assayed at 1, 3, 5, 7, and 14 days using the plate count method on LB agar. Commercial nisin A (1,000 U) (Cayman Chemical, Ann Arbor, MI, USA) was used as a positive control, and ddH_2_O was used as a negative control. All tests were performed in triplicate.

### Accession numbers.

The whole-genome sequence of B. toyonensis XIN-YC13 was deposited in the GenBank database with accession no. JABTXX000000000.1. The nucleotide sequence of the toyoncin gene cluster was deposited in the GenBank database under accession no. MT795152.
